# Effectiveness of social media-assisted course on learning self-efficacy

**DOI:** 10.1038/s41598-024-60724-0

**Published:** 2024-05-02

**Authors:** Jiaying Hu, Yicheng Lai, Xiuhua Yi

**Affiliations:** 1 College of Art and Design, Nanning Normal University, Nanning, 530000 Guangxi China; 2 Graduate School of Techno Design, Kookmin University, Seoul, 02707 Korea; 3 College of Music, Hubei Normal University, Huangshi, 435000 Hubei China

**Keywords:** Social media, Learning self-efficacy, Design students, Online learning, Design professional, Psychology, Human behaviour

## Abstract

The social media platform and the information dissemination revolution have changed the thinking, needs, and methods of students, bringing development opportunities and challenges to higher education. This paper introduces social media into the classroom and uses quantitative analysis to investigate the relation between design college students’ learning self-efficacy and social media for design students, aiming to determine the effectiveness of social media platforms on self-efficacy. This study is conducted on university students in design media courses and is quasi-experimental, using a randomized pre-test and post-test control group design. The study participants are 73 second-year design undergraduates. Independent samples t-tests showed that the network interaction factors of social media had a significant impact on college students learning self-efficacy. The use of social media has a significant positive predictive effect on all dimensions of learning self-efficacy. Our analysis suggests that using the advantages and value of online social platforms, weakening the disadvantages of the network, scientifically using online learning resources, and combining traditional classrooms with the Internet can improve students' learning self-efficacy.

## Introduction

Social media is a way of sharing information, ideas, and opinions with others one. It can be used to create relationships between people and businesses. Social media has changed the communication way, it’s no longer just about talking face to face but also using a digital platform such as Facebook or Twitter. Today, social media is becoming increasingly popular in everyone's lives, including students and researchers^1^. Social media provides many opportunities for learners to publish their work globally, bringing many benefits to teaching and learning. The publication of students' work online has led to a more positive attitude towards learning and increased achievement and motivation. Other studies report that student online publications or work promote reflection on personal growth and development and provide opportunities for students to imagine more clearly the purpose of their work^2^. In addition, learning environments that include student publications allow students to examine issues differently, create new connections, and ultimately form new entities that can be shared globally^3,4^.

Learning self-efficacy is a belief that you can learn something new. It comes from the Latin word “self” and “efficax” which means efficient or effective. Self-efficacy is based on your beliefs about yourself, how capable you are to learn something new, and your ability to use what you have learned in real-life situations. This concept was first introduced by Bandura (1977), who studied the effects of social reinforcement on children’s learning behavior. He found that when children were rewarded for their efforts they would persist longer at tasks that they did not like or had low interest in doing. Social media, a ubiquitous force in today's digital age, has revolutionized the way people interact and share information. With the rise of social media platforms, individuals now have access to a wealth of online resources that can enhance their learning capabilities. This access to information and communication has also reshaped the way students approach their studies, potentially impacting their learning self-efficacy. Understanding the role of social media in shaping students' learning self-efficacy is crucial in providing effective educational strategies that promote healthy learning and development^5^. Unfortunately, the learning curve for the associated metadata base modeling methodologies and their corresponding computer-aided software engineering (CASE) tools have made it difficult for students to grasp. Addressing this learning issue examined the effect of this MLS on the self-efficacy of learning these topics^6^. Bates et al.^7^ hypothesize a mediated model in which a set of antecedent variables influenced students’ online learning self-efficacy which, in turn, affected student outcome expectations, mastery perceptions, and the hours spent per week using online learning technology to complete learning assignments for university courses. Shen et al.^8^ through exploratory factor analysis identifies five dimensions of online learning self-efficacy: (a) self-efficacy to complete an online course (b) self-efficacy to interact socially with classmates (c) self-efficacy to handle tools in a Course Management System (CMS) (d) self-efficacy to interact with instructors in an online course, and (e) self-efficacy to interact with classmates for academic purposes. Chiu^9^ established a model for analyzing the mediating effect that learning self-efficacy and social self-efficacy have on the relationship between university students’ perceived life stress and smartphone addiction. Kim et al.^10^ study was conducted to examine the influence of learning efficacy on nursing students' self-confidence. The objective of Paciello et al.^11^ was to identify self-efficacy configurations in different domains (i.e., emotional, social, and self-regulated learning) in a sample of university students using a person-centered approach. The role of university students’ various conceptions of learning in their academic self-efficacy in the domain of physics is initially explored^12^. Kumar et al.^13^ investigated factors predicting students’ behavioral intentions towards the continuous use of mobile learning. Other influential work includes^14^.

Many studies have focused on social networking tools such as Facebook and MySpace^15,16^. Teachers are concerned that the setup and use of social media apps take up too much of their time, may have plagiarism and privacy issues, and contribute little to actual student learning outcomes; they often consider them redundant or simply not conducive to better learning outcomes^17^. Cao et al.^18^ proposed that the central questions in addressing the positive and negative pitfalls of social media on teaching and learning are whether the use of social media in teaching and learning enhances educational effectiveness, and what motivates university teachers to use social media in teaching and learning. Maloney et al.^3^ argued that social media can further improve the higher education teaching and learning environment, where students no longer access social media to access course information. Many studies in the past have shown that the use of modern IT in the classroom has increased over the past few years; however, it is still limited mainly to content-driven use, such as accessing course materials, so with the emergence of social media in students’ everyday lives^2^, we need to focus on developing students’ learning self-efficacy so that they can This will enable students to 'turn the tables and learn to learn on their own. Learning self-efficacy is considered an important concept that has a powerful impact on learning outcomes^19,20^.

Self-efficacy for learning is vital in teaching students to learn and develop healthily and increasing students' beliefs in the learning process^21^. However, previous studies on social media platforms such as Twitter and Weibo as curriculum support tools have not been further substantiated or analyzed in detail. In addition, the relationship between social media, higher education, and learning self-efficacy has not yet been fully explored by researchers in China. Our research aims to fill this gap in the topic. Our study explored the impact of social media on the learning self-efficacy of Chinese college students. Therefore, it is essential to explore the impact of teachers' use of social media to support teaching and learning on students' learning self-efficacy. Based on educational theory and methodological practice, this study designed a teaching experiment using social media to promote learning self-efficacy by posting an assignment for post-course work on online media to explore the actual impact of social media on university students’ learning self-efficacy. This study examines the impact of a social media-assisted course on university students' learning self-efficacy to explore the positive impact of a social media-assisted course.

## Theoretical background

### Social media

Social media has different definitions. Mayfield (2013) first introduced the concept of social media in his book-what is social media? The author summarized the six characteristics of social media: openness, participation, dialogue, communication, interaction, and communication. Mayfield^22^ shows that social media is a kind of new media. Its uniqueness is that it can give users great space and freedom to participate in the communication process. Jen (2020) also suggested that the distinguishing feature of social media is that it is “aggregated”. Social media provides users with an interactive service to control their data and information and collaborate and share information^2^. Social media offers opportunities for students to build knowledge and helps them actively create and share information^23^. Millennial students are entering higher education institutions and are accustomed to accessing and using data from the Internet. These individuals go online daily for educational or recreational purposes. Social media is becoming increasingly popular in the lives of everyone, including students and researchers^1^. A previous study has shown that millennials use the Internet as their first source of information and Google as their first choice for finding educational and personal information^24^. Similarly, many institutions encourage teachers to adopt social media applications^25^. Faculty members have also embraced social media applications for personal, professional, and pedagogical purposes^17^.

Social networks allow one to create a personal profile and build various networks that connect him/her to family, friends, and other colleagues. Users use these sites to stay in touch with their friends, make plans, make new friends, or connect with someone online. Therefore, extending this concept, these sites can establish academic connections or promote cooperation and collaboration in higher education classrooms^2^. This study defines social media as an interactive community of users' information sharing and social activities built on the technology of the Internet. Because the concept of social media is broad, its connotations are consistent. Research shows that Meaning and Linking are the two key elements that make up social media existence. Users and individual media outlets generate social media content and use it as a platform to get it out there. Social media distribution is based on social relationships and has a better platform for personal information and relationship management systems. Examples of social media applications include Facebook, Twitter, MySpace, YouTube, Flickr, Skype, Wiki, blogs, Delicious, Second Life, open online course sites, SMS, online games, mobile applications, and more^18^. Ajjan and Hartshorne^2^ investigated the intentions of 136 faculty members at a US university to adopt Web 2.0 technologies as tools in their courses. They found that integrating Web 2.0 technologies into the classroom learning environment effectively increased student satisfaction with the course and improved their learning and writing skills. His research focused on improving the perceived usefulness, ease of use, compatibility of Web 2.0 applications, and instructor self-efficacy. The social computing impact of formal education and training and informal learning communities suggested that learning web 2.0 helps users to acquire critical competencies, and promotes technological, pedagogical, and organizational innovation, arguing that social media has a variety of learning content^26^. Users can post digital content online, enabling learners to tap into tacit knowledge while supporting collaboration between learners and teachers. Cao and Hong^27^ investigated the antecedents and consequences of social media use in teaching among 249 full-time and part-time faculty members, who reported that the factors for using social media in teaching included personal social media engagement and readiness, external pressures; expected benefits; and perceived risks. The types of Innovators, Early adopters, Early majority, Late majority, Laggards, and objectors. Cao et al.^18^ studied the educational effectiveness of 168 teachers' use of social media in university teaching. Their findings suggest that social media use has a positive impact on student learning outcomes and satisfaction. Their research model provides educators with ideas on using social media in the education classroom to improve student performance. Maqableh et al.^28^ investigated the use of social networking sites by 366 undergraduate students, and they found that weekly use of social networking sites had a significant impact on student's academic performance and that using social networking sites had a significant impact on improving students' effective time management, and awareness of multitasking. All of the above studies indicate the researcher’s research on social media aids in teaching and learning. All of these studies indicate the positive impact of social media on teaching and learning.

### Learning self-efficacy

For the definition of concepts related to learning self-efficacy, scholars have mainly drawn on the idea proposed by Bandura^29^ that defines self-efficacy as “the degree to which people feel confident in their ability to use the skills they possess to perform a task”. Self-efficacy is an assessment of a learner’s confidence in his or her ability to use the skills he or she possesses to complete a learning task and is a subjective judgment and feeling about the individual’s ability to control his or her learning behavior and performance^30^. Liu^31^ has defined self-efficacy as the belief’s individuals hold about their motivation to act, cognitive ability, and ability to perform to achieve their goals, showing the individual's evaluation and judgment of their abilities. Zhang (2015) showed that learning efficacy is regarded as the degree of belief and confidence that expresses the success of learning. Yan^32^ showed the extent to which learning self-efficacy is viewed as an individual. Pan^33^ suggested that learning self-efficacy in an online learning environment is a belief that reflects the learner's ability to succeed in the online learning process. Kang^34^ believed that learning self-efficacy is the learner's confidence and belief in his or her ability to complete a learning task. Huang^35^ considered self-efficacy as an individual’s self-assessment of his or her ability to complete a particular task or perform a specific behavior and the degree of confidence in one’s ability to achieve a specific goal. Kong^36^ defined learning self-efficacy as an individual’s judgment of one’s ability to complete academic tasks.

Based on the above analysis, we found that scholars' focus on learning self-efficacy is on learning behavioral efficacy and learning ability efficacy, so this study divides learning self-efficacy into learning behavioral efficacy and learning ability efficacy for further analysis and research^37,38^. Search the CNKI database and ProQuest Dissertations for keywords such as “design students’ learning self-efficacy”, “design classroom self-efficacy”, “design learning self-efficacy”, and other keywords. There are few relevant pieces of literature about design majors. Qiu^39^ showed that mobile learning-assisted classroom teaching can control the source of self-efficacy from many aspects, thereby improving students’ sense of learning efficacy and helping middle and lower-level students improve their sense of learning efficacy from all dimensions. Yin and Xu^40^ argued that the three elements of the network environment—“learning content”, “learning support”, and “social structure of learning”—all have an impact on university students’ learning self-efficacy. Duo et al.^41^ recommend that learning activities based on the mobile network learning community increase the trust between students and the sense of belonging in the learning community, promote mutual communication and collaboration between students, and encourage each other to stimulate their learning motivation. In the context of social media applications, self-efficacy refers to the level of confidence that teachers can successfully use social media applications in the classroom^18^. Researchers have found that self-efficacy is related to social media applications^42^. Students had positive experiences with social media applications through content enhancement, creativity experiences, connectivity enrichment, and collaborative engagement^26^. Students who wish to communicate with their tutors in real-time find social media tools such as web pages, blogs, and virtual interactions very satisfying^27^. Overall, students report their enjoyment of different learning processes through social media applications; simultaneously, they show satisfactory tangible achievement of tangible learning outcomes^18^. According to Bandura's 'triadic interaction theory’, Bian^43^ and Shi^44^ divided learning self-efficacy into two main elements, basic competence, and control, where basic competence includes the individual's sense of effort, competence, the individual sense of the environment, and the individual's sense of control over behavior. The primary sense of competence includes the individual's Sense of effort, competence, environment, and control over behavior. In this study, learning self-efficacy is divided into Learning behavioral efficacy and Learning ability efficacy. Learning behavioral efficacy includes individuals' sense of effort, environment, and control; learning ability efficacy includes individuals' sense of ability, belief, and interest.

**Figure 1:**
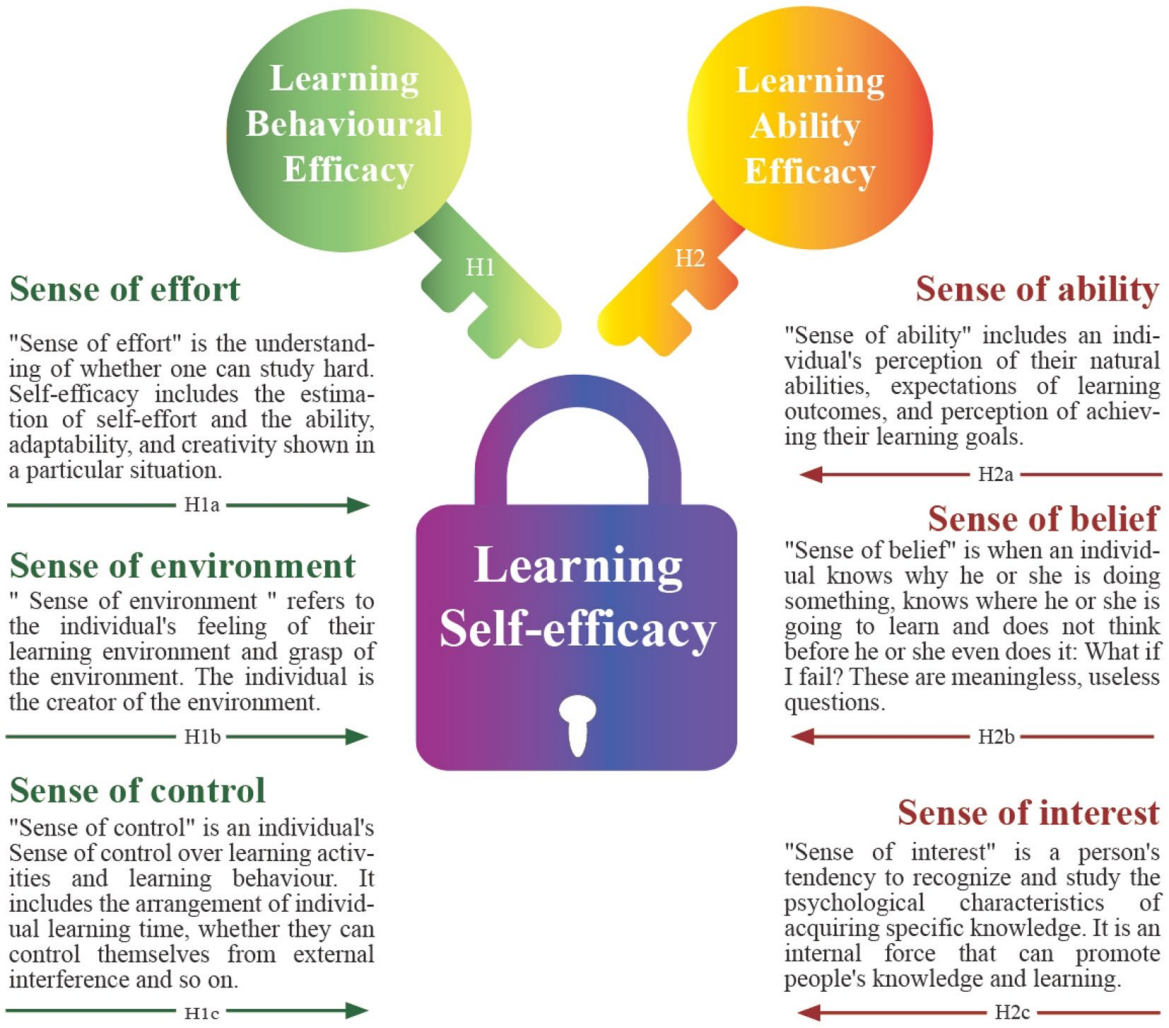
Learning self-efficacy research structure in this paper.

In Fig. 1, learning self-efficacy includes learning behavior efficacy and learning ability efficacy, in which the learning behavior efficacy is determined by the sense of effort, the sense of environment, the sense of control, and the learning ability efficacy is determined by the sense of ability, sense of belief, sense of interest. “Sense of effort” is the understanding of whether one can study hard. Self-efficacy includes the estimation of self-effort and the ability, adaptability, and creativity shown in a particular situation. One with a strong sense of learning self-efficacy thinks they can study hard and focus on tasks^44^. “Sense of environment” refers to the individual’s feeling of their learning environment and grasp of the environment. The individual is the creator of the environment. A person’s feeling and grasp of the environment reflect the strength of his sense of efficacy to some extent. A person with a shared sense of learning self-efficacy is often dissatisfied with his environment, but he cannot do anything about it. He thinks the environment can only dominate him. A person with a high sense of learning self-efficacy will be more satisfied with his school and think that his teachers like him and are willing to study in school^44^. “Sense of control” is an individual’s sense of control over learning activities and learning behavior. It includes the arrangement of individual learning time, whether they can control themselves from external interference, and so on. A person with a strong sense of self-efficacy will feel that he is the master of action and can control the behavior and results of learning. Such a person actively participates in various learning activities. When he encounters difficulties in learning, he thinks he can find a way to solve them, is not easy to be disturbed by the outside world, and can arrange his own learning time. The opposite is the sense of losing control of learning behavior^44^. “Sense of ability” includes an individual’s perception of their natural abilities, expectations of learning outcomes, and perception of achieving their learning goals. A person with a high sense of learning self-efficacy will believe that he or she is brighter and more capable in all areas of learning; that he or she is more confident in learning in all subjects. In contrast, people with low learning self-efficacy have a sense of powerlessness. They are self-doubters who often feel overwhelmed by their learning and are less confident that they can achieve the appropriate learning goals^44^. “Sense of belief” is when an individual knows why he or she is doing something, knows where he or she is going to learn, and does not think before he or she even does it: What if I fail? These are meaningless, useless questions. A person with a high sense of learning self-efficacy is more robust, less afraid of difficulties, and more likely to reach their learning goals. A person with a shared sense of learning self-efficacy, on the other hand, is always going with the flow and is uncertain about the outcome of their learning, causing them to fall behind. “Sense of interest” is a person's tendency to recognize and study the psychological characteristics of acquiring specific knowledge. It is an internal force that can promote people's knowledge and learning. It refers to a person's positive cognitive tendency and emotional state of learning. A person with a high sense of self-efficacy in learning will continue to concentrate on studying and studying, thereby improving learning. However, one with low learning self-efficacy will have psychology such as not being proactive about learning, lacking passion for learning, and being impatient with learning. The elements of learning self-efficacy can be quantified and detailed in the following Fig. 1.

## Methods

### Research participants

All the procedures were conducted in adherence to the guidelines and regulations set by the institution. Prior to initiating the study, informed consent was obtained in writing from the participants, and the Institutional Review Board for Behavioral and Human Movement Sciences at Nanning Normal University granted approval for all protocols.

Two parallel classes are pre-selected as experimental subjects in our study, one as the experimental group and one as the control group. Social media assisted classroom teaching to intervene in the experimental group, while the control group did not intervene. When selecting the sample, it is essential to consider, as far as possible, the shortcomings of not using randomization to select or assign the study participants, resulting in unequal experimental and control groups. When selecting the experimental subjects, classes with no significant differences in initial status and external conditions, i.e. groups with homogeneity, should be selected. Our study finally decided to select a total of 44 students from Class 2021 Design 1 and a total of 29 students from Class 2021 Design 2, a total of 74 students from Nanning Normal University, as the experimental subjects. The former served as the experimental group, and the latter served as the control group. 73 questionnaires are distributed to measure before the experiment, and 68 are returned, with a return rate of 93.15%. According to the statistics, there were 8 male students and 34 female students in the experimental group, making a total of 44 students (mirrors the demographic trends within the humanities and arts disciplines from which our sample was drawn); there are 10 male students and 16 female students in the control group, making a total of 26 students, making a total of 68 students in both groups. The sample of those who took the course were mainly sophomores, with a small number of first-year students and juniors, which may be related to the nature of the subject of this course and the course system offered by the university. From the analysis of students' majors, liberal arts students in the experimental group accounted for the majority, science students and art students accounted for a small part. In contrast, the control group had more art students, and liberal arts students and science students were small. In the daily self-study time, the experimental and control groups are 2–3 h. The demographic information of research participants is shown in Table 1.

**Table 1:** The demographic characteristics of the participants.

Category	Division	Group (%)	
		Experimental group (n = 42)	Control group (n = 26)
Gender	Male	8(19.05)	10(38.46)
	Female	34(80.95)	16(61.54)
Grade	Freshman	1(2.38)	2(7.69)
	Sophomore	40(95.24)	22(84.62)
	Junior	1(2.38)	2(7.69)
Major	liberal arts	25(59.52)	10(38.46)
	science	6(14.29)	3(11.54)
	Art	11(26.19)	13(50.00)
Average self-study time per day	none	2.19 ± 0.94	2.58 ± 0.81

## Research procedure

Firstly, the ADDIE model is used for the innovative design of the teaching method of the course. The number of students in the experimental group was 44, 8 male and 35 females; the number of students in the control group was 29, 10 male and 19 females. Secondly, the classes are targeted at students and applied. Thirdly, the course for both the experimental and control classes is a convenient and practice-oriented course, with the course title “Graphic Design and Production”, which focuses on learning the graphic design software Photoshop. The course uses different cases to explain in detail the process and techniques used to produce these cases using Photoshop, and incorporates practical experience as well as relevant knowledge in the process, striving to achieve precise and accurate operational steps; at the end of the class, the teacher assigns online assignments to be completed on social media, allowing students to post their edited software tutorials online so that students can master the software functions. The teacher assigns online assignments to be completed on social media at the end of the lesson, allowing students to post their editing software tutorials online so that they can master the software functions and production skills, inspire design inspiration, develop design ideas and improve their design skills, and improve students' learning self-efficacy through group collaboration and online interaction. Fourthly, pre-tests and post-tests are conducted in the experimental and control classes before the experiment. Fifthly, experimental data are collected, analyzed, and summarized.

We use a questionnaire survey to collect data. Self-efficacy is a person’s subjective judgment on whether one can successfully perform a particular achievement. American psychologist Albert Bandura first proposed it. To understand the improvement effect of students’ self-efficacy after the experimental intervention, this work questionnaire was referenced by the author from “Self-efficacy” “General Perceived Self Efficacy Scale” (General Perceived Self Efficacy Scale) German psychologist Schwarzer and Jerusalem (1995) and “Academic Self-Efficacy Questionnaire”, a well-known Chinese scholar Liang^45^.  The questionnaire content is detailed in the supplementary information. A pre-survey of the questionnaire is conducted here. The second-year students of design majors collected 32 questionnaires, eliminated similar questions based on the data, and compiled them into a formal survey scale. The scale consists of 54 items, 4 questions about basic personal information, and 50 questions about learning self-efficacy. The Likert five-point scale is the questionnaire used in this study. The answers are divided into “completely inconsistent", “relatively inconsistent”, “unsure”, and “relatively consistent”. The five options of “Completely Meet” and “Compliant” will count as 1, 2, 3, 4, and 5 points, respectively. Divided into a sense of ability (Q5–Q14), a sense of effort (Q15–Q20), a sense of environment (Q21–Q28), a sense of control (Q29–Q36), a sense of Interest (Q37–Q45), a sense of belief (Q46–Q54). To demonstrate the scientific effectiveness of the experiment, and to further control the influence of confounding factors on the experimental intervention. This article thus sets up a control group as a reference. Through the pre-test and post-test in different periods, comparison of experimental data through pre-and post-tests to illustrate the effects of the intervention.

Reliability indicates the consistency of the results of a measurement scale (See Table 2). It consists of intrinsic and extrinsic reliability, of which intrinsic reliability is essential. Using an internal consistency reliability test scale, a Cronbach's alpha coefficient of reliability statistics greater than or equal to 0.9 indicates that the scale has good reliability, 0.8–0.9 indicates good reliability, 7–0.8 items are acceptable. Less than 0.7 means to discard some items in the scale^46^. This study conducted a reliability analysis on the effects of the related 6-dimensional pre-test survey to illustrate the reliability of the questionnaire.

**Table 2:** Cronbach's α coefficients for learning self-efficacy.

Dimension	Item	Cronbach's α coefficient	
		Pre-test	Post-test
Sense of ability	10 (Q5–Q14)	0.919	0.898
Sense of effort	6 (Q15–Q20)	0.839	0.888
Sense of environment	8 (Q21–Q28)	0.848	0.886
Sense of control	8 (Q29–Q36)	0.865	0.889
Sense of interest	9(Q37–Q45)	0.852	0.900
Sense of belief	9(Q46–Q54)	0.889	0.893
Total	50	0.958	0.970

From the Table 2, the Cronbach alpha coefficients for the pre-test, sense of effort, sense of environment, sense of control, sense of interest, sense of belief, and the total questionnaire, were 0.919, 0.839, 0.848, 0.865, 0.852, 0.889 and 0.958 respectively. The post-test Cronbach alpha coefficients were 0.898, 0.888, 0.886, 0.889, 0.900, 0.893 and 0.970 respectively. The Cronbach alpha coefficients were all greater than 0.8, indicating a high degree of reliability of the measurement data.

The validity, also known as accuracy, reflects how close the measurement result is to the “true value”. Validity includes structure validity, content validity, convergent validity, and discriminative validity. Because the experiment is a small sample study, we cannot do any specific factorization. KMO and Bartlett sphericity test values are an important part of structural validity. Indicator, general validity evaluation (KMO value above 0.9, indicating very good validity; 0.8–0.9, indicating good validity; 0.7–0.8 validity is good; 0.6–0.7 validity is acceptable; 0.5–0.6 means poor validity; below 0.45 means that some items should be abandoned.

Table 3 shows that the KMO values of ability, effort, environment, control, interest, belief, and the total questionnaire are 0.911, 0.812, 0.778, 0.825, 0.779, 0.850, 0.613, and the KMO values of the post-test are respectively. The KMO values are 0.887, 0.775, 0.892, 0.868, 0.862, 0.883, 0.715. KMO values are basically above 0.8, and all are greater than 0.6. This result indicates that the validity is acceptable, the scale has a high degree of reasonableness, and the valid data.

**Table 3:** KMO and Bartlett's test.

	KMO		Approx. Chi-Square	
	Pre-test	Post-test	Pre-test	Post-test
Sense of ability	0.911	0.887	387.729	303.293
Sense of effort	0.812	0.775	161.652	237.346
Sense of environment	0.778	0.892	236.539	240.650
Sense of control	0.825	0.868	237.359	260.983
Sense of interest	0.779	0.862	232.309	306.515
Sense of belief	0.850	0.883	315.921	274.995
Total	0.613	0.715	3070.805	2838.310

In the graphic design and production (professional design course), we will learn the practical software with cases. After class, we will share knowledge on the self-media platform. We will give face-to-face computer instruction offline from 8:00 to 11:20 every Wednesday morning for 16 weeks. China's top online sharing platform (APP) is Tik Tok, micro-blog (Micro Blog) and Xiao hong shu. The experiment began on September 1, 2022, and conducted the pre-questionnaire survey simultaneously. At the end of the course, on January 6, 2023, the post questionnaire survey was conducted. A total of 74 questionnaires were distributed in this study, recovered 74 questionnaires. After excluding the invalid questionnaires with incomplete filling and wrong answers, 68 valid questionnaires were obtained, with an effective rate of 91%, meeting the test requirements. Then, use the social science analysis software SPSS Statistics 26 to analyze the data: (1) descriptive statistical analysis of the dimensions of learning self-efficacy; (2) Using correlation test to analyze the correlation between learning self-efficacy and the use of social media; (3) This study used a comparative analysis of group differences to detect the influence of learning self-efficacy on various dimensions of social media and design courses. For data processing and analysis, use the spss26 version software and frequency statistics to create statistics on the basic situation of the research object and the basic situation of the use of live broadcast. The reliability scale analysis (internal consistency test) and use Bartlett's sphericity test to illustrate the reliability and validity of the questionnaire and the individual differences between the control group and the experimental group in demographic variables (gender, grade, Major, self-study time per day) are explained by cross-analysis (chi-square test). In the experimental group and the control group, the pre-test, post-test, before-and-after test of the experimental group and the control group adopt independent sample T-test and paired sample T-test to illustrate the effect of the experimental intervention (The significance level of the test is 0.05 two-sided).

## Results and discussion

### Comparison of pre-test and post-test between groups

To study whether the data of the experimental group and the control group are significantly different in the pre-test and post-test mean of sense of ability, sense of effort, sense of environment, sense of control, sense of interest, and sense of belief. The research for this situation uses an independent sample T-test and an independent sample. The test needs to meet some false parameters, such as normality requirements. Generally passing the normality test index requirements are relatively strict, so it can be relaxed to obey an approximately normal distribution. If there is serious skewness distribution, replace it with the nonparametric test. Variables are required to be continuous variables. The six variables in this study define continuous variables. The variable value information is independent of each other. Therefore, we use the independent sample T-test.

From the Table 4, a pre-test found that there was no statistically significant difference between the experimental group and the control group at the 0.05 confidence level (*p* > 0.05) for perceptions of sense of ability, sense of effort, sense of environment, sense of control, sense of interest, and sense of belief. Before the experiment, the two groups of test groups have the same quality in measuring self-efficacy. The experimental class and the control class are homogeneous groups. Table 5 shows the independent samples t-test for the post-test, used to compare the experimental and control groups on six items, including the sense of ability, sense of effort, sense of environment, sense of control, sense of interest, and sense of belief.

**Table 4:** Comparison of pre-test between Experimental and Control groups on learning self-efficacy.

Dimension	Group(M ± SD)		*t*	p
	Experimental group (n = 42)	Control group (n = 26)		
Sense of ability	3.41 ± 0.55	3.47 ± 0.73	− 0.358	0.721
Sense of effort	3.31 ± 0.65	3.46 ± 0.74	− 0.828	0.411
Sense of environment	3.47 ± 0.44	3.52 ± 0.69	− 0.302	0.764
Sense of control	3.27 ± 0.52	3.38 ± 0.59	− 0.764	0.448
Sense of interest	3.25 ± 0.59	3.40 ± 0.65	− 0.938	0.352
Sense of belief	3.58 ± 0.58	3.49 ± 0.65	0.597	0.553
Total	3.38 ± 0.40	3.45 ± 0.60	− 0.554	0.582

**Table 5:** Comparison of post-test between Experimental and Control groups on learning self-efficacy.

Dimension	Group(M ± SD)		*t*	p
	Experimental group (n = 42)	Control group (n = 26)		
Sense of ability	3.91 ± 0.51	3.43 ± 0.73	3.177	0.002**
Sense of effort	3.88 ± 0.66	3.31 ± 0.94	2.911	0.005**
Sense of environment	3.95 ± 0.61	3.58 ± 0.62	2.451	0.017*
Sense of control	3.76 ± 0.67	3.31 ± 0.78	2.524	0.014*
Sense of interest	3.87 ± 0.61	3.39 ± 0.77	2.842	0.006**
Sense of belief	4.04 ± 0.52	3.56 ± 0.65	3.377	0.001**
Total	3.90 ± 0.48	3.43 ± 0.66	3.422	0.001**

The experimental and control groups have statistically significant scores (*p* < 0.05) for sense of ability, sense of effort, sense of environment, sense of control, sense of interest, and sense of belief, and the experimental and control groups have statistically significant scores (t = 3.177, *p* = 0.002) for a sense of competence. (t = 3.177, *p* = 0.002) at the 0.01 level, with the experimental group scoring significantly higher (3.91 ± 0.51) than the control group (3.43 ± 0.73). The experimental group and the control group showed significance for the perception of effort at the 0.01 confidence level (t = 2.911, *p* = 0.005), with the experimental group scoring significantly higher (3.88 ± 0.66) than the control group scoring significantly higher (3.31 ± 0.94). The experimental and control groups show significance at the 0.05 level (t = 2.451, *p* = 0.017) for the sense of environment, with the experimental group scoring significantly higher (3.95 ± 0.61) than the control group scoring significantly higher (3.58 ± 0.62). The experimental and control groups showed significance for sense of control at the 0.05 level of significance (t = 2.524, *p* = 0.014), and the score for the experimental group (3.76 ± 0.67) would be significantly higher than the score for the control group (3.31 ± 0.78). The experimental and control groups showed significance at the 0.01 level for sense of interest (t = 2.842, *p* = 0.006), and the experimental group's score (3.87 ± 0.61) would be significantly higher than the control group's score (3.39 ± 0.77). The experimental and control groups showed significance at the 0.01 level for the sense of belief (t = 3.377, *p* = 0.001), and the experimental group would have scored significantly higher (4.04 ± 0.52) than the control group (3.56 ± 0.65). Therefore, we can conclude that the experimental group's post-test significantly affects the mean scores of sense of ability, sense of effort, sense of environment, sense of control, sense of interest, and sense of belief. A social media-assisted course has a positive impact on students' self-efficacy.

### Comparison of pre-test and post-test of each group

The paired-sample T-test is an extension of the single-sample T-test. The purpose is to explore whether the means of related (paired) groups are significantly different. There are four standard paired designs: (1) Before and after treatment of the same subject Data, (2) Data from two different parts of the same subject, (3) Test results of the same sample with two methods or instruments, 4. Two matched subjects receive two treatments, respectively. This study belongs to the first type, the 6 learning self-efficacy dimensions of the experimental group and the control group is measured before and after different periods.

Paired t-tests is used to analyze whether there is a significant improvement in the learning self-efficacy dimension in the experimental group after the experimental social media-assisted course intervention. In Table 6, we can see that the six paired data groups showed significant differences (*p* < 0.05) in the pre and post-tests of sense of ability, sense of effort, sense of environment, sense of control, sense of interest, and sense of belief. There is a level of significance of 0.01 (t = − 4.540, *p* = 0.000 < 0.05) before and after the sense of ability, the score after the sense of ability (3.91 ± 0.51), and the score before the Sense of ability (3.41 ± 0.55). The level of significance between the pre-test and post-test of sense of effort is 0.01 (t = − 4.002, *p* = 0.000). The score of the sense of effort post-test (3.88 ± 0.66) will be significantly higher than the average score of the sense of effort pre-test (3.31 ± 0.659). The significance level between the pre-test and post-test Sense of environment is 0.01 (t = − 3.897, *p* = 0.000). The average score for post- Sense of environment (3.95 ± 0.61) will be significantly higher than that of sense of environment—the average score of the previous test (3.47 ± 0.44). The average value of a post- sense of control (3.76 ± 0.67) will be significantly higher than the average of the front side of the Sense of control value (3.27 ± 0.52). The sense of interest pre-test and post-test showed a significance level of 0.01 (− 4.765, *p* = 0.000), and the average value of Sense of interest post-test was 3.87 ± 0.61. It would be significantly higher than the average value of the Sense of interest (3.25 ± 0.59), the significance between the pre-test and post-test of belief sensing is 0.01 level (t = − 3.939, *p* = 0.000). Thus, the average value of a post-sense of belief (4.04 ± 0.52) will be significantly higher than that of a pre-sense of belief Average value (3.58 ± 0.58). After the experimental group’s post-test, the scores for the Sense of ability, effort, environment, control, interest, and belief before the comparison experiment increased significantly. This result has a significant improvement effect. Table 7 shows that the control group did not show any differences in the pre and post-tests using paired t-tests on the dimensions of learning self-efficacy such as sense of ability, sense of effort, sense of environment, sense of control, sense of interest, and sense of belief (*p* > 0.05). It shows no experimental intervention for the control group, and it does not produce a significant effect.

**Table 6:** Comparison between pre-test and post-test of experimental group on learning self-efficacy.

Dimension	Experimental group(M ± SD)		m	*t*	p
	Pre-test	Post-test			
Sense of ability	3.41 ± 0.55	3.91 ± 0.51	− 0.50	− 4.540	0.000***
Sense of effort	3.31 ± 0.65	3.88 ± 0.66	− 0.57	− 4.002	0.000***
Sense of environment	3.47 ± 0.44	3.95 ± 0.61	− 0.48	− 3.897	0.000***
Sense of control	3.27 ± 0.52	3.76 ± 0.67	− 0.49	− 3.422	0.001**
Sense of interest	3.25 ± 0.59	3.87 ± 0.61	− 0.62	− 4.765	0.000***
Sense of belief	3.58 ± 0.58	4.04 ± 0.52	− 0.46	− 3.939	0.000***
Total	3.38 ± 0.40	3.90 ± 0.48	− 0.52	− 5.452	0.000***

**Table 7:** Comparison between pre-test and post-test of control group on learning self-efficacy.

Dimension	Control group		m	*t*	p
	Pre-test	Post-test			
Sense of ability	3.47 ± 0.73	3.43 ± 0.73	0.03	0.159	0.875
Sense of effort	3.46 ± 0.74	3.31 ± 0.94	0.14	0.511	0.614
Sense of environment	3.52 ± 0.69	3.58 ± 0.62	− 0.06	− 0.293	0.772
Sense of control	3.38 ± 0.59	3.31 ± 0.78	0.07	0.313	0.757
Sense of interest	3.40 ± 0.65	3.39 ± 0.77	0.01	0.039	0.969
Sense of belief	3.49 ± 0.65	3.56 ± 0.65	− 0.06	− 0.297	0.769
Total	3.45 ± 0.60	3.43 ± 0.66	0.02	0.106	0.916

## Conclusion

The purpose of this study aims to explore the impact of social media use on college students' learning self-efficacy, examine the changes in the elements of college students' learning self-efficacy before and after the experiment, and make an empirical study to enrich the theory. This study developed an innovative design for course teaching methods using the ADDIE model. The design process followed a series of model rules of analysis, design, development, implementation, and evaluation, as well as conducted a descriptive statistical analysis of the learning self-efficacy of design undergraduates. Using questionnaires and data analysis, the correlation between the various dimensions of learning self-efficacy is tested. We also examined the correlation between the two factors, and verifies whether there was a causal relationship between the two factors.

Based on prior research and the results of existing practice, a learning self-efficacy is developed for university students and tested its reliability and validity. The scale is used to pre-test the self-efficacy levels of the two subjects before the experiment, and a post-test of the self-efficacy of the two groups is conducted. By measuring and investigating the learning self-efficacy of the study participants before the experiment, this study determined that there was no significant difference between the experimental group and the control group in terms of sense of ability, sense of effort, sense of environment, sense of control, sense of interest, and sense of belief. Before the experiment, the two test groups had homogeneity in measuring the dimensionality of learning self-efficacy. During the experiment, this study intervened in social media assignments for the experimental group. The experiment used learning methods such as network assignments, mutual aid communication, mutual evaluation of assignments, and group discussions. After the experiment, the data analysis showed an increase in learning self-efficacy in the experimental group compared to the pre-test. With the test time increased, the learning self-efficacy level of the control group decreased slightly. It shows that social media can promote learning self-efficacy to a certain extent. This conclusion is similar to Cao et al.^18^, who suggested that social media would improve educational outcomes.

We have examined the differences between the experimental and control group post-tests on six items, including the sense of ability, sense of effort, sense of environment, sense of control, sense of interest, and sense of belief. This result proves that a social media-assisted course has a positive impact on students' learning self-efficacy. Compared with the control group, students in the experimental group had a higher interest in their major. They showed that they liked to share their learning experiences and solve difficulties in their studies after class. They had higher motivation and self-directed learning ability after class than students in the control group. In terms of a sense of environment, students in the experimental group were more willing to share their learning with others, speak boldly, and participate in the environment than students in the control group.

The experimental results of this study showed that the experimental group showed significant improvement in the learning self-efficacy dimensions after the experimental intervention in the social media-assisted classroom, with significant increases in the sense of ability, sense of effort, sense of environment, sense of control, sense of interest and sense of belief compared to the pre-experimental scores. This result had a significant improvement effect. Evidence that a social media-assisted course has a positive impact on students' learning self-efficacy. Most of the students recognized the impact of social media on their learning self-efficacy, such as encouragement from peers, help from teachers, attention from online friends, and recognition of their achievements, so that they can gain a sense of achievement that they do not have in the classroom, which stimulates their positive perception of learning and is more conducive to the awakening of positive effects. This phenomenon is in line with Ajjan and Hartshorne^2^. They argue that social media provides many opportunities for learners to publish their work globally, which brings many benefits to teaching and learning. The publication of students' works online led to similar positive attitudes towards learning and improved grades and motivation. This study also found that students in the experimental group in the post-test controlled their behavior, became more interested in learning, became more purposeful, had more faith in their learning abilities, and believed that their efforts would be rewarded. This result is also in line with Ajjan and Hartshorne's (2008) indication that integrating Web 2.0 technologies into classroom learning environments can effectively increase students' satisfaction with the course and improve their learning and writing skills.

We only selected students from one university to conduct a survey, and the survey subjects were self-selected. Therefore, the external validity and generalizability of our study may be limited. Despite the limitations, we believe this study has important implications for researchers and educators. The use of social media is the focus of many studies that aim to assess the impact and potential of social media in learning and teaching environments. We hope that this study will help lay the groundwork for future research on the outcomes of social media utilization. In addition, future research should further examine university support in encouraging teachers to begin using social media and university classrooms in supporting social media (supplementary file 1).

The present study has provided preliminary evidence on the positive association between social media integration in education and increased learning self-efficacy among college students. However, several avenues for future research can be identified to extend our understanding of this relationship.

Firstly, replication studies with larger and more diverse samples are needed to validate our findings across different educational contexts and cultural backgrounds. This would enhance the generalizability of our results and provide a more robust foundation for the use of social media in teaching. Secondly, longitudinal investigations should be conducted to explore the sustained effects of social media use on learning self-efficacy. Such studies would offer insights into how the observed benefits evolve over time and whether they lead to improved academic performance or other relevant outcomes. Furthermore, future research should consider the exploration of potential moderators such as individual differences in students' learning styles, prior social media experience, and psychological factors that may influence the effectiveness of social media in education. Additionally, as social media platforms continue to evolve rapidly, it is crucial to assess the impact of emerging features and trends on learning self-efficacy. This includes an examination of advanced tools like virtual reality, augmented reality, and artificial intelligence that are increasingly being integrated into social media environments. Lastly, there is a need for research exploring the development and evaluation of instructional models that effectively combine traditional teaching methods with innovative uses of social media. This could guide educators in designing courses that maximize the benefits of social media while minimizing potential drawbacks.

In conclusion, the current study marks an important step in recognizing the potential of social media as an educational tool. Through continued research, we can further unpack the mechanisms by which social media can enhance learning self-efficacy and inform the development of effective educational strategies in the digital age.

### Supplementary Information

Supplementary Information.

## Data Availability

The data that support the findings of this study are available from the corresponding authors upon reasonable request. The data are not publicly available due to privacy or ethical restrictions.

## References

[1] Rasheed MI (2020). Usage of social media, student engagement, and creativity: The role of knowledge sharing behavior and cyberbullying. Comput. Educ..

[2] Ajjan H, Hartshorne R (2008). Investigating faculty decisions to adopt Web 2.0 technologies: Theory and empirical tests. Internet High. Educ..

[3] Maloney, E. J. What web 2.0 can teach us about learning. *The Chronicle of Higher Education* **53**, B26–B27 (2007).

[4] Ustun AB, Karaoglan-Yilmaz FG, Yilmaz R (2023). Educational UTAUT-based virtual reality acceptance scale: A validity and reliability study. Virtual Real..

[5] Schunk DH (1985). Self-efficacy and classroom learning. Psychol. Sch..

[6] Cheung W, Li EY, Yee LW (2003). Multimedia learning system and its effect on self-efficacy in database modeling and design: An exploratory study. Comput. Educ..

[7] Bates R, Khasawneh S (2007). Self-efficacy and college students’ perceptions and use of online learning systems. Comput. Hum. Behav..

[8] Shen D, Cho M-H, Tsai C-L, Marra R (2013). Unpacking online learning experiences: Online learning self-efficacy and learning satisfaction. Internet High. Educ..

[9] Chiu S-I (2014). The relationship between life stress and smartphone addiction on taiwanese university student: A mediation model of learning self-efficacy and social self-Efficacy. Comput. Hum. Behav..

[10] Kim S-O, Kang B-H (2016). The influence of nursing students’ learning experience, recognition of importance and learning self-efficacy for core fundamental nursing skills on their self-confidence. J. Korea Acad.-Ind. Coop. Soc..

[11] Paciello M, Ghezzi V, Tramontano C, Barbaranelli C, Fida R (2016). Self-efficacy configurations and wellbeing in the academic context: A person-centred approach. Pers. Individ. Differ..

[12] Suprapto N, Chang T-S, Ku C-H (2017). Conception of learning physics and self-efficacy among Indonesian University students. J. Balt. Sci. Educ..

[13] Kumar JA, Bervell B, Annamalai N, Osman S (2020). Behavioral intention to use mobile learning: Evaluating the role of self-efficacy, subjective norm, and WhatsApp use habit. IEEE Access.

[14] Fisk JE, Warr P (1996). Age-related impairment in associative learning: The role of anxiety, arousal and learning self-efficacy. Pers. Indiv. Differ..

[15] Pence HE (2007). Preparing for the real web generation. J. Educ. Technol. Syst..

[16] Hu J, Lee J, Yi X (2023). Blended knowledge sharing model in design professional. Sci. Rep..

[17] Moran, M., Seaman, J. & Tintikane, H. Blogs, wikis, podcasts and Facebook: How today’s higher education faculty use social media, vol. 22, 1–28 (Pearson Learning Solutions. Retrieved December, 2012).

[18] Cao Y, Ajjan H, Hong P (2013). Using social media applications for educational outcomes in college teaching: A structural equation analysis: Social media use in teaching. Br. J. Educ. Technol..

[19] Artino AR (2012). Academic self-efficacy: From educational theory to instructional practice. Perspect. Med. Educ..

[20] Pajares F (1996). Self-efficacy beliefs in academic settings. Rev. Educ. Res..

[21] Zhao, Z. *Classroom Teaching Design of Layout Design Based on Self Efficacy Theory* (Tianjin University of Technology and Education, 2021).

[22] Yılmaz FGK, Yılmaz R (2023). Exploring the role of sociability, sense of community and course satisfaction on students’ engagement in flipped classroom supported by facebook groups. J. Comput. Educ..

[23] Nguyen NP, Yan G, Thai MT (2013). Analysis of misinformation containment in online social networks. Comput. Netw..

[24] Connaway LS, Radford ML, Dickey TJ, Williams JDA, Confer P (2008). Sense-making and synchronicity: Information-seeking behaviors of millennials and baby boomers. Libri.

[25] Wankel, C., Marovich, M. & Stanaityte, J. *Cutting-edge social media approaches to business education : teaching with LinkedIn, Facebook, Twitter, Second Life, and blogs*. (Global Management Journal, 2010).

[26] Redecker, C., Ala-Mutka, K. & Punie, Y. Learning 2.0: The impact of social media on learning in Europe. *Policy brief. JRC Scientific and Technical Report. EUR JRC56958 EN*. Available from http://bit.ly/cljlpq [Accessed 6 th February 2011] **6** (2010).

[27] Cao Y, Hong P (2011). Antecedents and consequences of social media utilization in college teaching: A proposed model with mixed-methods investigation. Horizon.

[28] Maqableh M (2015). The impact of social media networks websites usage on students’ academic performance. Commun. Netw..

[29] Bandura A (1997). Self-Efficacy.

[30] Karaoglan-Yilmaz FG, Ustun AB, Zhang K, Yilmaz R (2023). Metacognitive awareness, reflective thinking, problem solving, and community of inquiry as predictors of academic self-efficacy in blended learning: A correlational study. Turk. Online J. Distance Educ..

[31] Liu, W. *Self-efficacy Level and Analysis of Influencing Factors on Non-English Major Bilingual University Students—An Investigation Based on Three* (Xinjiang Normal University, 2015).

[32] Yan, W. *Influence of College Students’ Positive Emotions on Learning Engagement and Academic Self-efficacy* (Shanghai Normal University, 2016).

[33] Pan, J. *Relational Model Construction between College Students’ Learning Self-efficacy and Their Online Autonomous Learning Ability* (Northeast Normal University, 2017).

[34] Kang, Y. *The Study on the Relationship Between Learning Motivation, Self-efficacy and Burnout in College Students* (Shanxi University of Finance and Economics, 2018).

[35] Huang, L. *A Study on the Relationship between Chinese Learning Efficacy and Learning Motivation of Foreign Students in China* (Huaqiao University, 2018).

[36] Kong, W. *Research on the Mediating Role of Undergraduates’ Learning Self-efficacy in the Relationship between Professional Identification and Learning Burnout* (Shanghai Normal University, 2019).

[37] Kuo TM, Tsai CC, Wang JC (2021). Linking web-based learning self-efficacy and learning engagement in MOOCs: The role of online academic hardiness. Internet High. Educ..

[38] Zhan, Y. *A Study of the Impact of Social Media Use and Dependence on Real-Life Social Interaction Among University Students* (Shanghai International Studies University, 2020).

[39] Qiu S (2017). A study on mobile learning to assist in developing English learning effectiveness among university students. J. Lanzhou Inst. Educ..

[40] Yin R, Xu D (2011). A study on the relationship between online learning environment and university students’ learning self-efficacy. E-educ. Res..

[41] Duo, Z., Zhao, W. & Ren, Y. A New paradigm for building mobile online learning communities: A perspective on the development of self-regulated learning efficacy among university students, in *Modern distance education* 10–17 (2019).

[42] Park SY, Nam M-W, Cha S-B (2012). University students’ behavioral intention to use mobile learning: Evaluating the technology acceptance model: Factors related to use mobile learning. Br. J. Educ. Technol..

[43] Bian, Y. *Development and application of the Learning Self-Efficacy Scale* (East China Normal University, 2003).

[44] Shi, X. *Between Life Stress and Smartphone Addiction on Taiwanese University Student* (Southwest University, 2010).

[45] Liang, Y. *Study On Achievement Goals、Attribution Styles and Academic Self-efficacy of Collage Students* (Central China Normal University, 2000).

[46] Qiu, H. *Quantitative Research and Statistical Analysis* (Chongqing University Press, 2013).

