# Supplementary material for: Effectiveness of social media-assisted course on learning self-efficacy
Source: Sci Rep. 2024 May 2;14:10112. doi: 10.1038/s41598-024-60724-0 (PMC11066128; doi:10.1038/s41598-024-60724-0)
Supplement: Supplementary file 1 — Supplementary Information. [file 41598_2024_60724_MOESM1_ESM.pdf]

Appendix

Self-Efficacy Questionnaire for College Students' Learning

Dear students,

Hello! I am a PhD student majoring in Education. Currently, I am conducting a survey on college students' learning ability. Thank you very much for taking the time to participate in this survey, as your feedback and involvement are crucial to my future research. There are no right or wrong answers, and please don't spend too much time on each question. Simply choose the option that best aligns with your current thoughts based on your actual circumstances. Thank you for your support, and I wish you a pleasant learning experience!

**Demographics**	
1. Your gender	A. Male
	B. Female
2.Your current grade	A. Freshman
	B. Sophomore
	C. Junior
	D. Senior
3. What is your major category?	A. Humanities
	B. Science
	C. Arts
4. How many hours do you typically spend studying per day?	A. No self-study
	B. less than 1 hour
	C. 2-3 hours
	D. 3-4 hours
	E. 5 hours or more

Please carefully read each statement below and assess the degree to which it aligns with your own thoughts. Note: it is not about agreeing with the statement, but rather whether you have had such thoughts yourself. Choose the corresponding option that best reflects your response on a five-point scale.

	QUESTION	Strongly disagree	Disagree	Neutral	Agree	Strongly agree
5	I believe I am capable of achieving good grades in my studies.	1	2	3	4	5

6	Compared to other classmates, I consider myself to have a stronger learning ability.	1	2	3	4	5
7	I believe I can apply what I have learned.	1	2	3	4	5
8	Even when facing setbacks in my learning, I can calmly analyze the mistakes I made.	1	2	3	4	5
9	Regardless of my academic performance, I never doubt my learning ability.	1	2	3	4	5
10	When contemplating an issue, I can connect the knowledge I have learned before and think comprehensively.	1	2	3	4	5
11	If I make an effort, I am always able to solve problems.	1	2	3	4	5
12	Even when others disagree with my viewpoints, I can still express my opinions effectively.	1	2	3	4	5
13	Overall, I am a confident individual.	1	2	3	4	5
14	I am confident in excelling in my major courses.	1	2	3	4	5
15	I put a lot of effort and dedication into my studies.	1	2	3	4	5
16	In my class, I am one of the more diligent students.	1	2	3	4	5
17	I attribute my academic success to my hard work.	1	2	3	4	5
18	I often think that if I don't study hard, I will lose competitiveness in future employment.	1	2	3	4	5
19	I enjoy challenging myself with more difficult learning tasks.	1	2	3	4	5
20	I want to study hard and improve my standing in the class.	1	2	3	4	5
21	I believe the learning atmosphere in my class is favorable.	1	2	3	4	5
22	I like the university I am currently attending.	1	2	3	4	5
23	I am satisfied with my major.	1	2	3	4	5
24	I am aware of the future career prospects in my major.	1	2	3	4	5
25	I believe that the teachers have a positive impression of me.	1	2	3	4	5
26	I can learn from those around me and gain insights that contribute to my personal growth.	1	2	3	4	5
27	In our class group, we often provide feedback to each other.	1	2	3	4	5
28	When faced with a wealth of online resources, I frequently organize and categorize them.	1	2	3	4	5
29	I have a reasonable time management plan for my studies.	1	2	3	4	5
30	I do not procrastinate and leave my assignments until the last minute.	1	2	3	4	5
31	I can concentrate on studying even in a noisy environment.	1	2	3	4	5
32	After being interrupted while studying, I can regain my focus.	1	2	3	4	5
33	I can pay attention in class.	1	2	3	4	5
34	When learning new course content, I often connect it with what I have previously learned in order to gain a deep understanding.	1	2	3	4	5
35	I regularly evaluate and provide feedback on my learning	1	2	3	4	5

	activities.					
36	I promptly correct and adjust any problems that arise during my learning process.	1	2	3	4	5
37	I generally find studying in college enjoyable.	1	2	3	4	5
38	As my studies progress, my interest in my major becomes stronger.	1	2	3	4	5
39	I rarely feel empty due to my efforts in learning.	1	2	3	4	5
40	I frequently visit the library after class to read books or journals related to my major.	1	2	3	4	5
41	I enjoy learning from others' professional knowledge shared online after class.	1	2	3	4	5
42	I am in a better mental state for learning in college compared to when I was in secondary school.	1	2	3	4	5
43	I often experience a strong sense of satisfaction due to my advantages in learning.	1	2	3	4	5
44	During my college studies, I often feel a sense of relief after resolving a problem.	1	2	3	4	5
45	When completing assigned learning tasks, I am interested in the learning activity itself.	1	2	3	4	5
46	I believe that learning valuable knowledge leads to personal growth and improvement.	1	2	3	4	5
47	When facing a learning task, as long as it is valuable, I take it seriously even if I don't enjoy it.	1	2	3	4	5
48	I believe that learning ability can be cultivated and improved.	1	2	3	4	5
49	I believe that college courses provide a lot of useful knowledge.	1	2	3	4	5
50	I believe that reading should be systematic, progressing from foundational books to more advanced ones.	1	2	3	4	5
51	I fully understand the value of learning.	1	2	3	4	5
52	During class, I always try to note down everything the teacher says, regardless of its significance.	1	2	3	4	5
53	Even if not required by the teacher, I willingly do extra practice to consolidate what I have learned.	1	2	3	4	5
54	After class, I often learn from others' shared professional knowledge online.	1	2	3	4	5
